# No genetic causal association between Alzheimer’s disease and osteoporosis: A bidirectional two-sample Mendelian randomization study

**DOI:** 10.3389/fnagi.2023.1090223

**Published:** 2023-01-25

**Authors:** Hongxin Hu, Jian Mei, Yuanqing Cai, Haiqi Ding, Susheng Niu, Wenming Zhang, Xinyu Fang

**Affiliations:** ^1^Department of Orthopedic Surgery, The First Affiliated Hospital of Fujian Medical University, Fuzhou, China; ^2^Department of Orthopaedic Surgery, National Regional Medical Center, Binhai Campus of the First Affiliated Hospital, Fujian Medical University, Fuzhou, China; ^3^Department of Orthopedic Surgery, Affiliated Hospital of Putian University, Putian, China; ^4^Department of Orthopaedics, The Second Affiliated Hospital of Xi’an Jiaotong University, Xi'an, Shaanxi, China; ^5^Key Laboratory of Orthopedics and Traumatology of Traditional Chinese Medicine and Rehabilitation Ministry of Education, Fujian University of Traditional Chinese Medicine, Fuzhou, China

**Keywords:** osteoporosis, Alzheimer’s disease, bone mineral density, Mendelian randomization, causal relationship

## Abstract

**Objective:**

Many observational studies have found an association between Alzheimer’s disease (AD) and osteoporosis. However, it is unclear whether there is causal genetic between osteoporosis and AD.

**Methods:**

A two-sample Mendelian randomization (MR) study was used to investigate whether there is a causal relationship between osteoporosis and AD. Genes for osteoporosis and AD were obtained from published the genome-wide association studies (GWAS). Single nucleotide polymorphisms (SNPs) with significant genome-wide differences (*p* < 5 × 10^−8^) and independent (*r*^2^ < 0.001) were selected, and SNPs with *F* ≥ 10 were further analyzed. Inverse variance weighted (IVW) was used to assess causality, and the results were reported as odds ratios (ORs). Subsequently, heterogeneity was tested using Cochran’s *Q* test, pleiotropy was tested using the MR–Egger intercept, and leave-one-out sensitivity analysis was performed to assess the robustness of the results.

**Results:**

Using the IVW method, MR Egger method, and median-weighted method, we found that the results showed no significant causal effect of osteoporosis at different sites and at different ages on AD, regardless of the removal of potentially pleiotropic SNPs. The results were similar for the opposite direction of causality. These results were confirmed to be reliable and stable by sensitivity analysis.

**Conclusion:**

This study found that there is no bidirectional causal relationship between osteoporosis and AD. However, they share similar pathogenesis and pathways.

## Introduction

The prevalence of Alzheimer’s disease (AD), a disease marked by progressive memory loss and cognitive deficits, is increasing (Broom et al., 2019; Breijyeh and Karaman, 2020). By 2050, the number of people with AD will exceed 100 million, imposing a massive economic burden on society (Sun et al., 2018). Osteoporosis is a systemic disease characterized by loss of bone that results in low bone mineral density and destruction of bone microarchitecture (Black et al., 2020). Osteoporosis can lead to fragility fractures, with approximately 9 million cases of fragility fractures due to osteoporosis worldwide each year, which further leads to decreased quality of life and an increased risk of death in patients (Che et al., 2023). According to the WHO definition, the current standard for clinical diagnosis and assessment of osteoporosis is mainly based on the measurement of bone mineral density (BMD; Cummings et al., 2002; Johnell et al., 2005).

It has been found that osteoporosis is twice as common in patients with AD as in patients with other neurological disorders, and AD patients’ risk of hip fracture is two to three times higher than that of people without AD (Tolppanen et al., 2013; Dengler-Crish and Elefteriou, 2019). A prospective study consisting of a stratified analysis of BMD found that the group with the lowest BMD was 3.48 times more likely to develop AD than group with the highest BMD (Zhou et al., 2011). Previous studies have shown that 60–80% of the risk of developing AD depends on genetic factors (Scheltens et al., 2021), and the heritability of osteoporosis is also 60–80% (Yang et al., 2020). To date, genome-wide association studies (GWAS) have identified more than 500 susceptibility loci associated with osteoporosis (Yang et al., 2020), and 533 SPNs and 126 genes have been linked to AD (Han et al., 2017). Osteoporosis and Alzheimer’s disease may share genetic and biological mechanisms, such as impaired cellular energy due to the effects of AKT (a serine–threonine kinase that is also known as protein kinase B, or PKB) on glucose uptake or defects in Wnt/β-linked protein signaling (Dengler-Crish and Elefteriou, 2019; Fehsel and Christl, 2022).

In addition, numerous of clinical observational studies have shown a strong association between osteoporosis and AD. Several prospective clinical studies have found a higher incidence of osteoporosis in AD patients than in healthy individuals, even after correcting for associated factors (Loskutova et al., 2009; Pu et al., 2020). A prospective clinical study by Zhou et al. (2011) found that patients with the lowest BMD had a 3.48-fold higher risk of AD than those with the highest BMD, and even after correcting for age, sex, and education, the risk was still elevated (2.68-fold).

Clarifying the causal relationship between osteoporosis and AD is crucial for prevention and treatment, but it is unclear whether such a causal relationship exists. Due to various confounding factors in clinical observational studies, the observations often fail to provide a convincing answer regarding a causal relationship between osteoporosis and AD. Mendelian randomization (MR) is a method used to assess whether there is a causal relationship between exposure factors and outcomes, as it uses genetic variants as instrumental variables (IVs) that are equally, randomly, and independently distributed during division (Emdin et al., 2017), and the assignment of genotypes is not influenced by age, sex, lifestyle, or environmental factors (Hartwig et al., 2017; Vaucher et al., 2018; Goto et al., 2020). The greatest benefit of MR compared to conventional clinical randomized controlled trials is that potential confounders are avoided (Didelez and Sheehan, 2007). Therefore, we used a two-sample MR design to assess the causal relationship between osteoporosis (as measured by BMD) and AD.

## Materials and Methods

### Study design and data source

**Figure 1:**
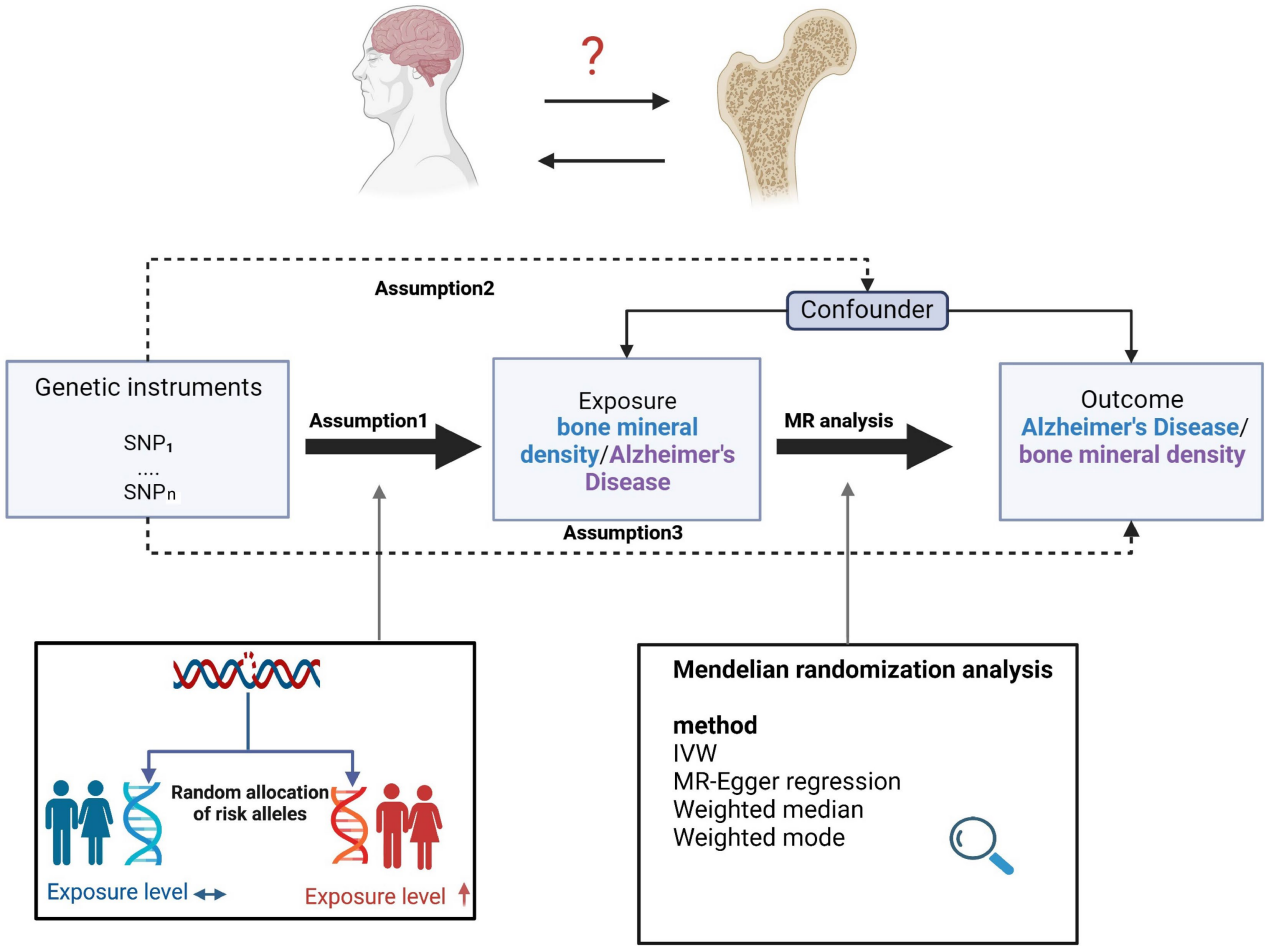
Overview of the study design and assumptions of the MR design. Assumption 1 is that the genetic variants proposed as instrumental variables are robustly associated with the risk factor of interest; assumption 2 is that the selected genetic variants are not associated with potential confounders; and assumption 3 is that the selected genetic variants affect the risk of the outcome solely through the risk factor and not through other pathways. The MR design reduces residual confounding and reverse causality, thereby strengthening causal inferences regarding exposure–outcome associations. The basis for this is that the genetic variants selected as instrumental variables to study altered exposure effects are randomly assigned at the time of conception and are therefore not susceptible to confounding by environmental factors and reverse causality. IVW, inverse variance weighted.

In this two-sample MR study, single nucleotide polymorphisms (SNPs) were used as IVs to determine the causal relationship between osteoporosis and AD using GWAS data. An overview of the study design and the hypotheses of the MR study are shown in Figure 1. Genes related to osteoporosis and AD were obtained from published GWAS, and data details are shown in Table 1.

**Table 1:** Data sources used in this study.

Exposures or outcome	Sample size (total or cases/controls)	Ancestry	Consortia	PubMed ID or URL
Alzheimer’s disease	21,982/41,944	European	open GWAS summary data	https://gwas.mrcieu.ac.uk/datasets/ieu-b-2/
Lumbar spine bone mineral density	28,498	European	GEFOS	26,367,794
Forearm bone mineral density	8,143	Mixed	GEFOS	26,367,794
Femoral neck bone mineral density	32,735	European	GEFOS	26,367,794
Heel bone mineral density (BMD)	265,627	European	UK Biobank	https://data.bris.ac.uk/data/dataset/pnoat8cxo0u52p6ynfaekeigi
Total body bone mineral density	56,284	European	GWAS meta-analysis	29,304,378
Total body bone mineral density (age 0–15)	11,807	European	GWAS meta-analysis	29,304,378
Total body bone mineral density (age 15–30)	4,180	European	GWAS meta-analysis	29,304,378
Total body bone mineral density (age 30–45)	10,062	European	GWAS meta-analysis	29,304,378
Total body bone mineral density (age 45–60)	18,805	European	GWAS meta-analysis	29,304,378
Total body bone mineral density (age over 60)	22,504	European	GWAS meta-analysis	29,304,378

### Instrumental variable selection

For MR analysis of osteoporosis and AD, we selected SNPs with significant genome-wide differences (*p* < 5 × 10^−8^) and tested their linkage disequilibrium (*r*^2^ < 0.001) as IVs; we then excluded SNPs with linkage disequilibrium. Finally, SNPs with *F* ≥ 10 were further analyzed.

### Statistical analysis

The random-effects inverse variance weighted (IVW) method was used to analyze the causal relationship between osteoporosis and AD. The causal effect of each SNP on the outcome was assessed by calculating the Wald ratio for each SNP, and the inverse variance of the SNP was used as the weight for meta-analysis to evaluate the joint causal effect. In addition, we used MR–Egger, the weighted median, and the weighted mode to assess the causal relationship between osteoporosis and AD. MR–Egger has low statistical power, so the focus is more on direction and effect (Luo et al., 2020; Cai et al., 2021). The weighted median provided a reliable Mendelian evaluation when 50% of the instrumental variables (instrument variables) were not valid (Wu et al., 2021). The odds ratio (OR) and 95% confidence interval (CI) were used to assess the relative risk due to the presence of the disease of interest. We used MR–Egger regression and IVW methods to test for heterogeneity among the selected SNPs and assessed the effect of heterogeneity using Cochrane’s Q statistic. In addition, we used the MR–Egger regression method to test for potential horizontal pleiotropy and performed a leave-one-out sensitivity analysis to assess the validity and stability of the MR results.

All data analyses were performed using the R package “two-sample MR” in R language (version 3.6.1) software. *p* < 0.05 was statistically significant. The data used in this study were publicly available and therefore did not require ethical approval for their use.

## Results

### Effect of AD on BMD at different sites

#### Results before removal of potentially pleiotropic SNPs

**Figure 2:**
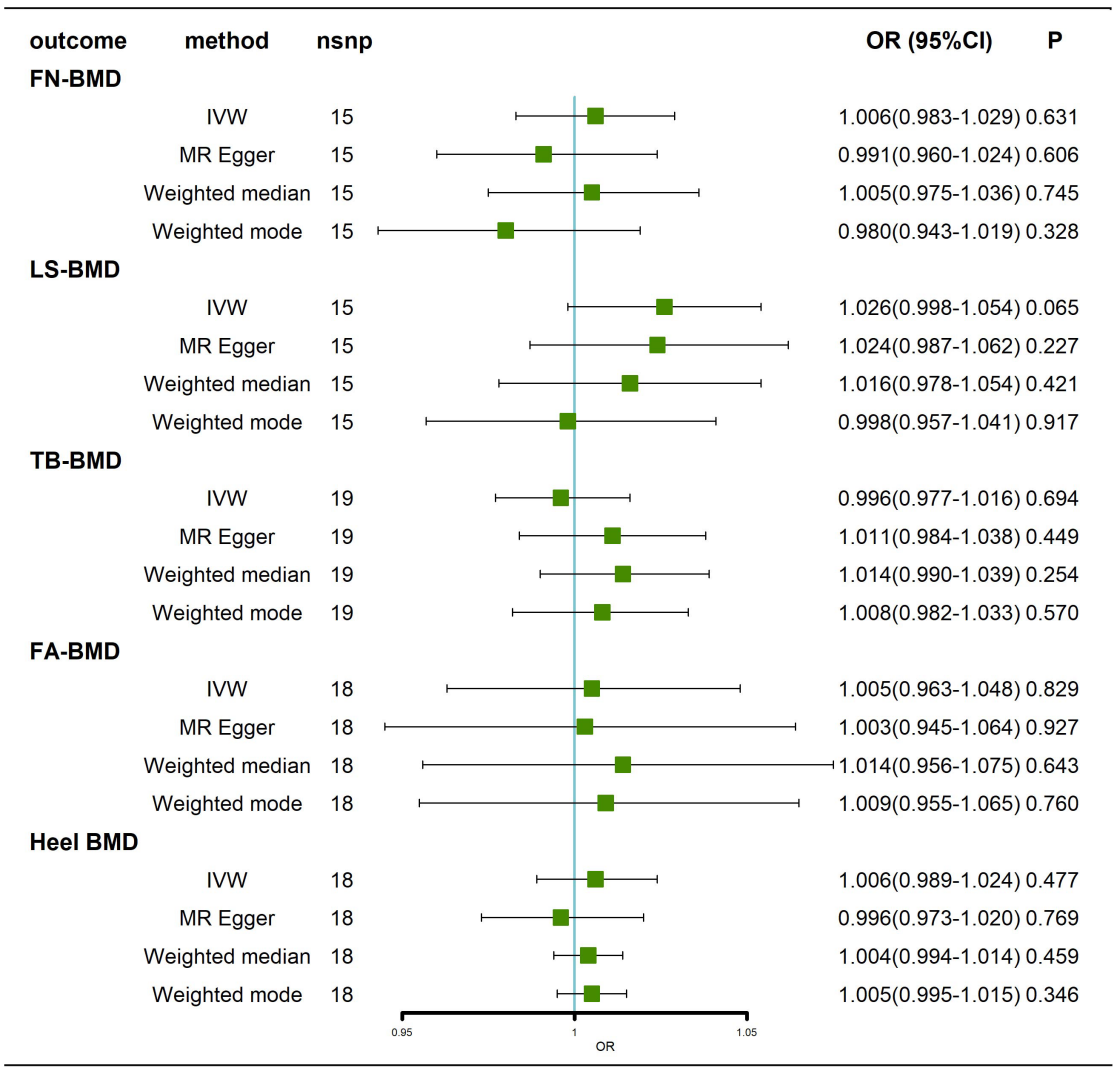
Causal effects of AD on BMD at different sites before removal of potentially pleiotropic SNPs. Odds ratios are expressed per 1-SD increase in genetically determined AD. AD: Alzheimer’s disease; FN-BMD: femoral neck bone mineral density; LS-BMD: lumbar spine bone mineral density; TB-BMD: total body bone mineral density; FA-BMD: forearm bone mineral density; Heel BMD: heel bone mineral density; nsnp, number of single nucleotide polymorphisms; CI, confidence interval.

Regarding the effect of AD on BMD at different sites, all IVW and MR–Egger methods failed to show any causal relationship of AD on BMD at different sites (Figure 2; Supplementary Figure 1). Cochran’s Q test showed no heterogeneity, except for Heel-BMD (femoral neck bone mineral density, FN-BMD, *Q* = 3.2917, *p* = 0.997; lumbar spine bone mineral density, LS-BMD, *Q* = 10.0841 *p* = 0.687; total body bone mineral density, TB-BMD, *Q* = 20.0115, *p* = 0.274; forearm bone mineral density, FA-BMD, *Q* = 15.7196, *p* = 0.473; heel bone mineral density, Heel-BMD, *Q* = 100.0997, *p* = 3.31 × 10^−14^; Supplementary Table 1; Supplementary Figures 2A-E). The results of the horizontal pleiotropy test showed no directional pleiotropy (FN-BMD, intercept = 0.0047, *p* = 0.221; LS-BMD, intercept = 0.0005, *p* = 0.907; TB-BMD, intercept = −0.0041, *p* = 0.153; FA-BMD, intercept = 0.0006, *p* = 0.930; Heel–BMD, intercept = 0.0033, *p* = 0.252; Supplementary Table 1). The results of the weighted median analysis were interpreted according to Nazarzadeh et al. (2020), and showed no causal effect of AD on BMD at different sites (Figure 2). Finally, to assess whether these results were influenced by a single SNP, we performed a leave-one-out sensitivity test, which showed that the causal effect of AD on BMD at different sites did not significantly fluctuate with any single SNP deletion (Supplementary Figures 2F–J). In summary, our results showed that there was no significant causal effect of AD on BMD at different sites.

#### Results after removal of potentially pleiotropic SNPs

**Figure 3:**
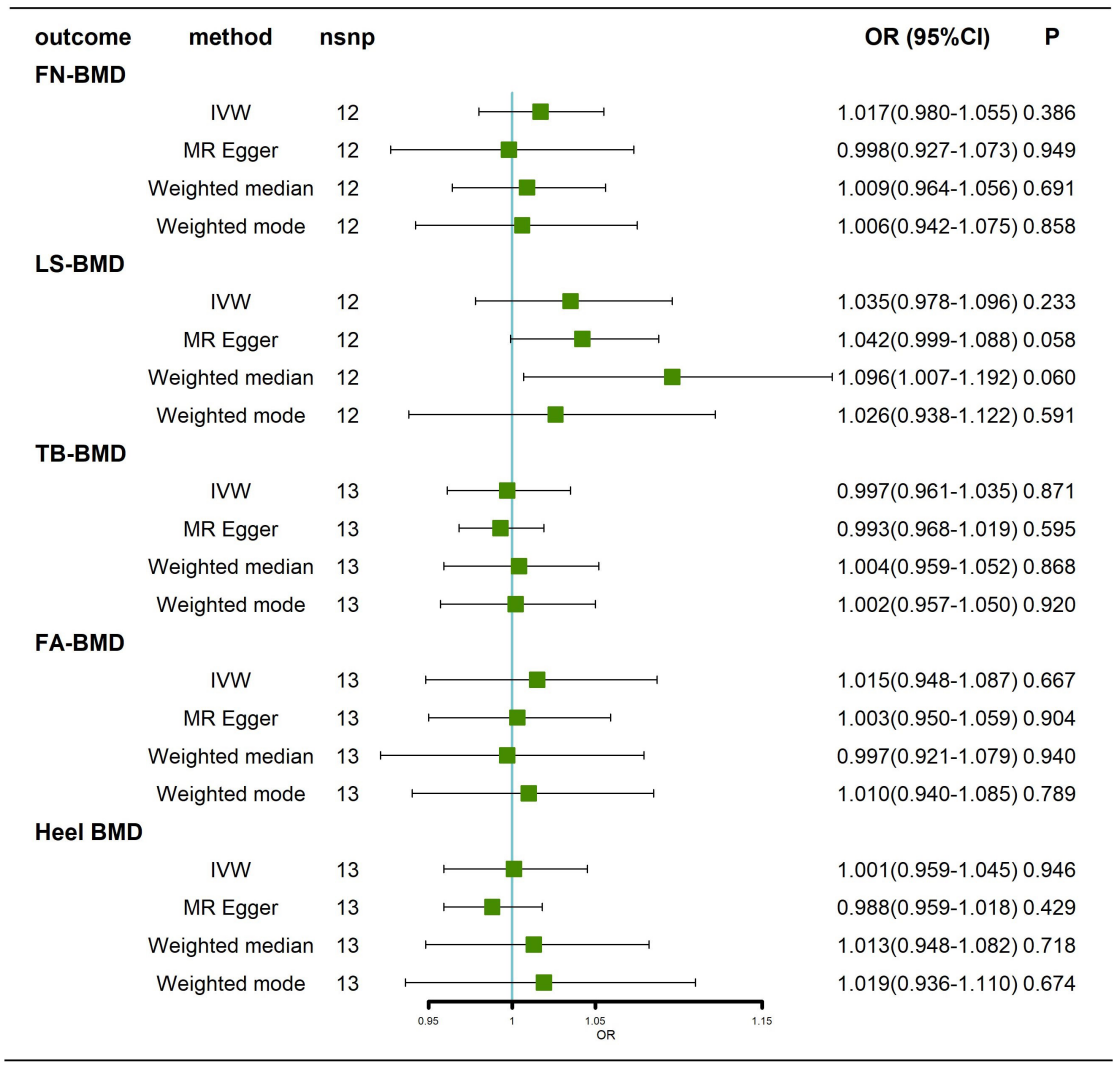
Causal effects of AD on BMD at different sites after removal of potentially pleiotropic SNPs. AD: Alzheimer’s disease; FN-BMD: femoral neck bone mineral density; LS-BMD: Lumbar spine bone mineral density; TB-BMD: total body bone mineral density; FA-BMD: forearm bone mineral density; Heel-BMD: heel bone mineral density; nsnp, number of single nucleotide polymorphisms; CI, confidence interval.

Traits association analysis (Table 2) showed that SNPs (rs34665982, rs1582763, rs3740688, rs7412, rs1081105, rs12151021, rs147711004) of AD-related genes were associated with high cholesterol, coronary artery disease and multiple potential confounders (leukocytes, lymphocytes, neutrophil count, BMI, heel bone density, inflammatory bowel disease, hemoglobin concentration, apolipoprotein B, C-reactive protein, waist circumference, LDL, etc.). After removal of pleiotropic SNPs, all IVW and weighted median methods showed similar findings (Figure 3; Supplementary Figure 3).

**Table 2:** The reported traits of selected SNP searched in phenoscanner.

SNP	Gene	Trait(s)
rs34665982	HLA-DRB1	Inflammatory bowel disease, White blood cell count, Hemoglobin concentration
rs1582763	MS4A4E	Alzheimer’s disease, Neutrophil count, Heel bone mineral density
rs3740688	SPI1	Alzheimer’s disease, Body mass index, Nervous feelings
rs7412	APOE	Alzheimer’s disease, APOB apolipoprotein B, Cholesterol total, Coronary artery disease
rs1081105	APOC1	Alzheimer’s disease, C-reactive protein, Self-reported high cholesterol, Waist circumference
rs12151021	ABCA7	Alzheimer’s disease, Lymphocyte count, Red cell distribution width
rs147711004	NECTIN2	Illnesses of mother: Alzheimer’s disease or dementia, Low density lipoprotein, Coronary artery disease
rs679515	CR1	Alzheimer’s disease
rs6733839	BIN1	Alzheimer’s disease
rs114812713	OARD1	Alzheimer’s disease
rs9381563	CD2AP	Alzheimer’s disease
rs11767557	EPHA1	Alzheimer’s disease
rs867230	CLU	Alzheimer’s disease
rs73223431	PTK2B	Alzheimer’s disease
rs11257242	RP11-138I18.2	Alzheimer’s disease
rs3851179	RNU6-560P	Alzheimer’s disease
rs12590654	SLC24A4	Alzheimer’s disease
rs72654445	APOC1	Alzheimer’s disease
rs111278137	CEACAM16	Alzheimer’s disease
rs139136389	APOC1	Alzheimer’s disease
rs150685845	TRAPPC6A	Alzheimer’s disease

### Effect of AD on BMD at different ages

#### Results before removal of potentially pleiotropic SNPs

**Figure 4:**
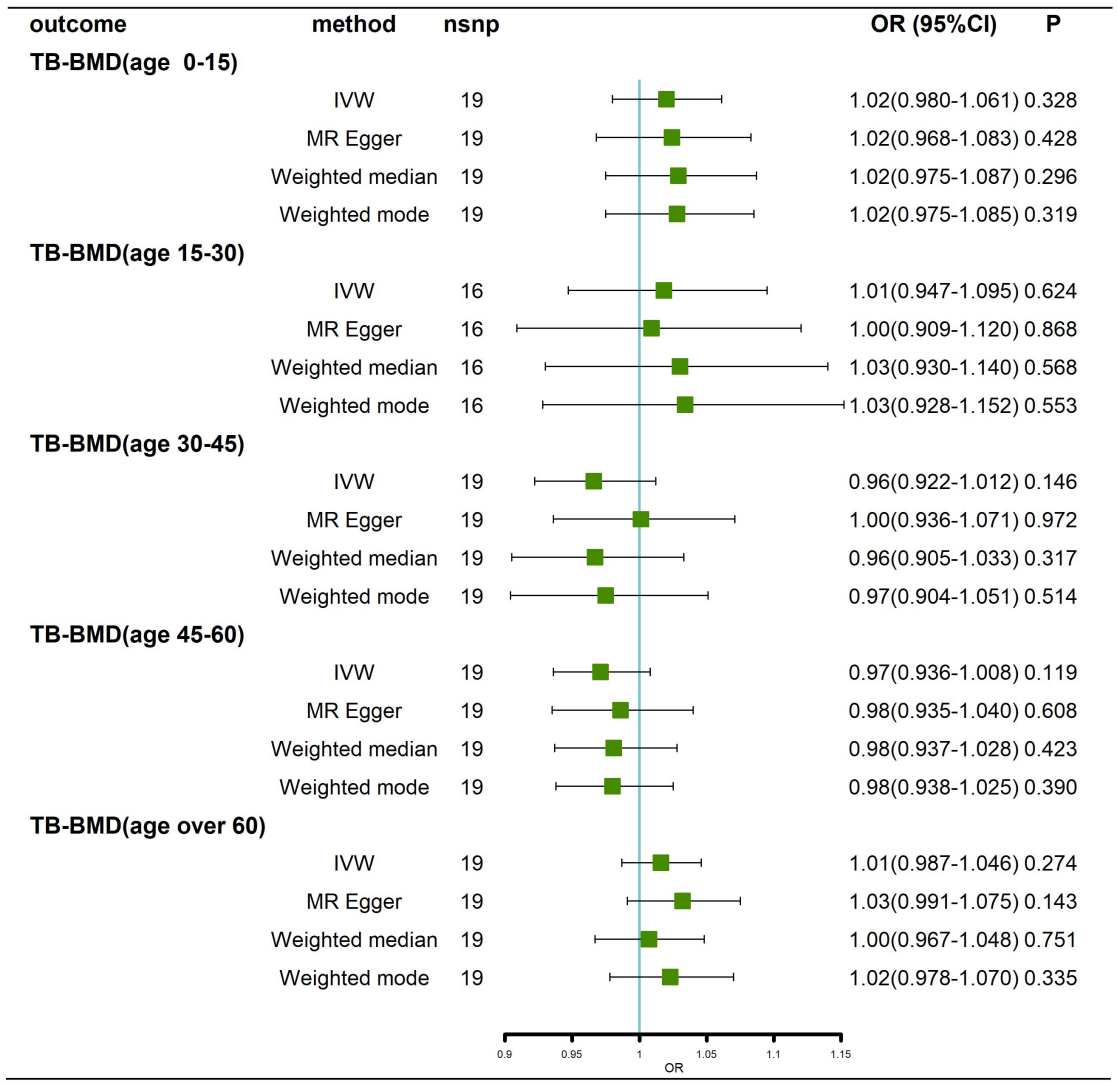
Causal effects of AD on BMD in different age groups before removal of potentially pleiotropic SNPs. AD: Alzheimer’s disease; TB-BMD: total body bone mineral density; SNP, single nucleotide polymorphism; IVW, inverse variance weighted; nsnp, number of single nucleotide polymorphisms; CI, confidence interval.

Briefly, we did not find any significant causal effect of AD on BMD at different ages, whether by the IVW method, MR–Egger, weighted median or weighted mode (Figure 4; Supplementary Figure 4). Next, we performed tests for heterogeneity and horizontal pleiotropy, which showed no heterogeneity (*p* > 0.05; Supplementary Table 2; Supplementary Figures 5A-E) and no directional pleiotropy (*p* > 0.05; Supplementary Table 2). Finally, we performed a leave-one-out sensitivity test, which showed that the causal effect of AD on BMD at different ages did not fluctuate significantly in the absence of any single SNP (Supplementary Figures 5F–J). In summary, our results show that there is no significant causal effect of AD on BMD at different ages.

#### Results after removal of potentially pleiotropic SNPs

**Figure 5:**
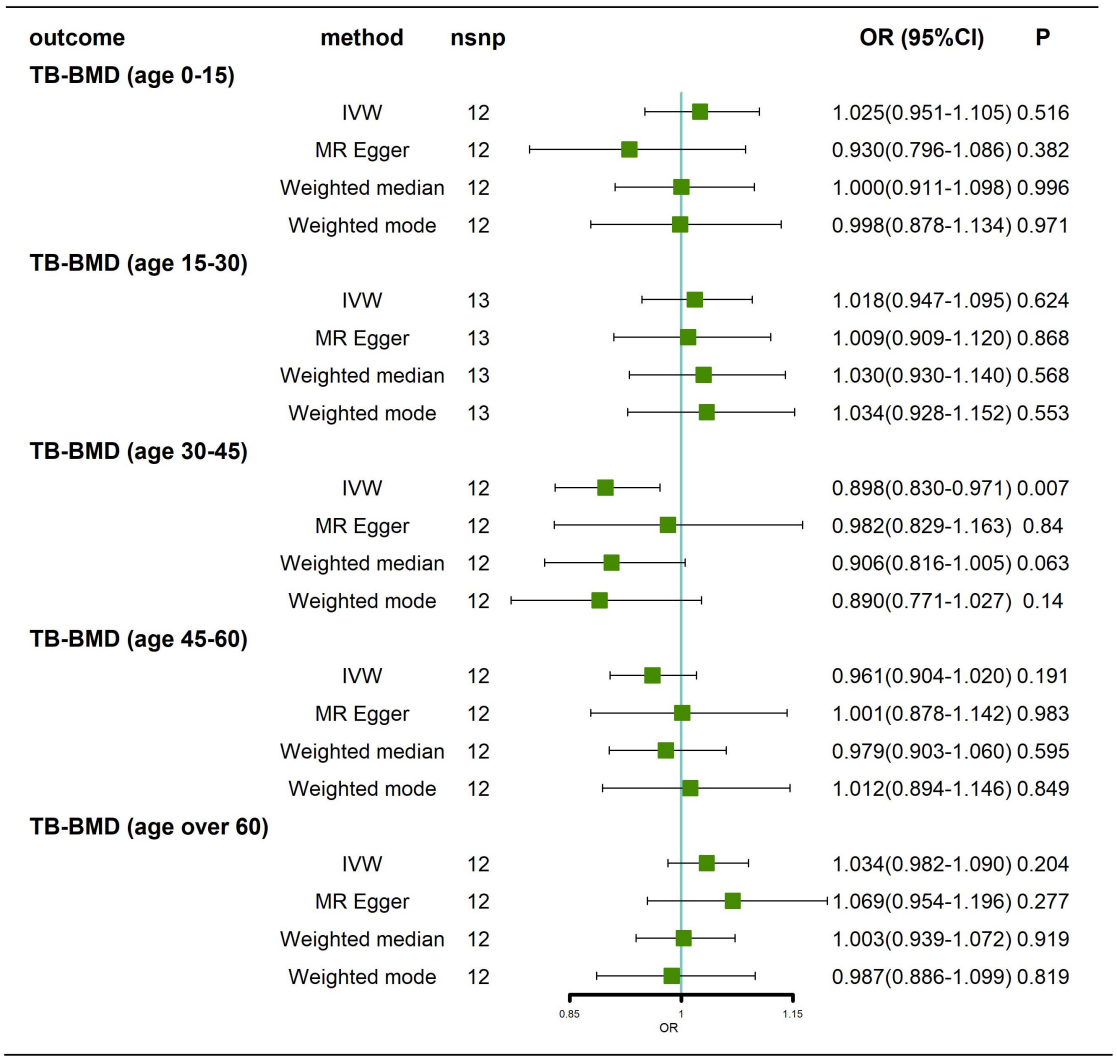
Causal effects of AD on BMD in different age groups after removal of potentially pleiotropic SNPs. AD: Alzheimer’s disease; TB-BMD: total body bone mineral density; SNP, single nucleotide polymorphism; IVW, inverse variance weighted; nsnp, number of single nucleotide polymorphisms; CI, confidence interval.

Traits association analysis (Table 2) identified some pleiotropic SNPs, and after these SNPs were removed, the results still showed that there was no causal effect of AD on BMD at different ages (Figure 5; Supplementary Figure 6).

### Effects of BMD at different sites on AD

**Figure 6:**
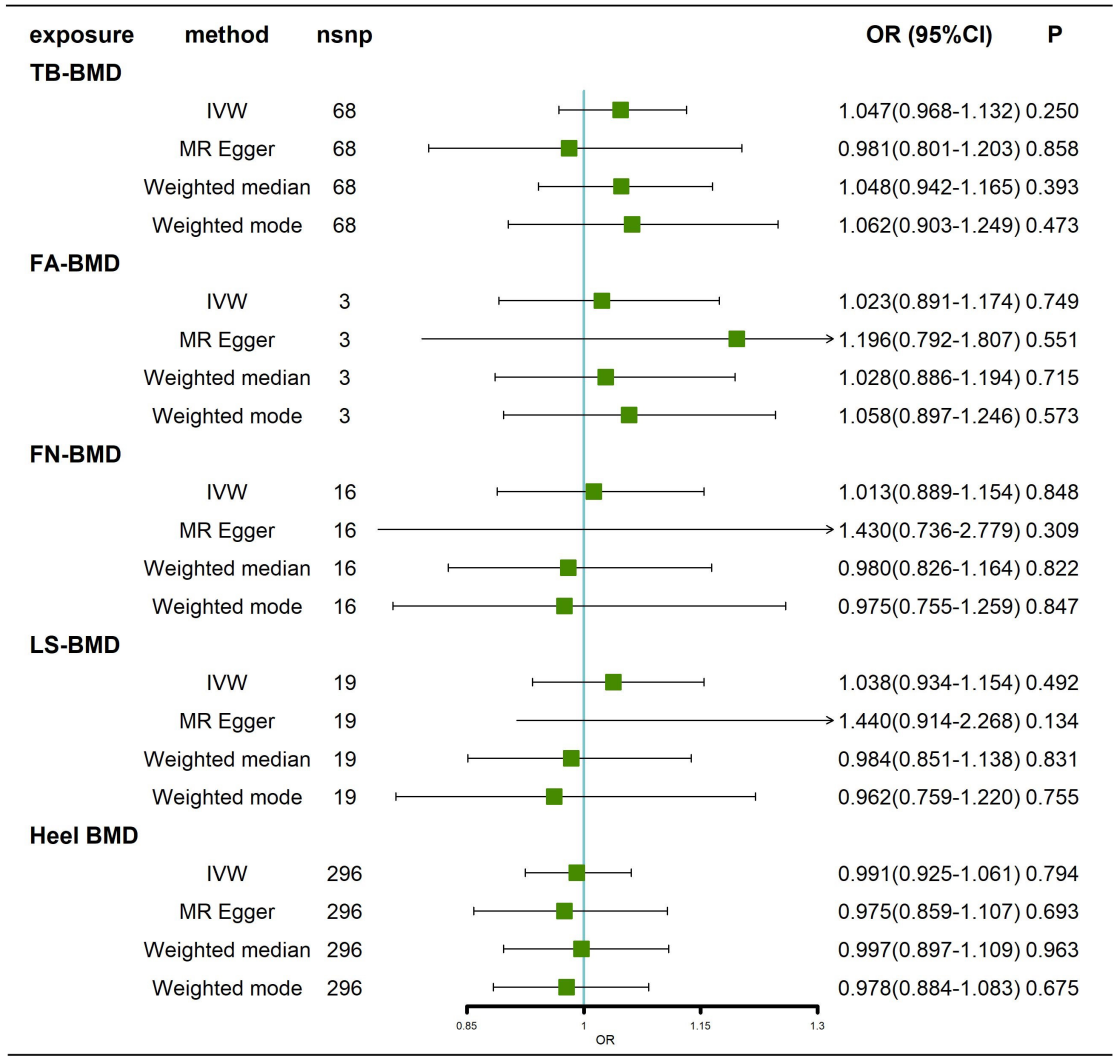
Causal effects of BMD at different sites on AD. AD: Alzheimer’s disease; TB-BMD: total body bone mineral density; SNP, single nucleotide polymorphism; IVW, inverse variance weighted; nsnp, number of single nucleotide polymorphisms; CI, confidence interval.

We obtained 68,3,16,19 and 296 SNPs from GWASs for TB-BMD, FA-BMD, FN-BMD, LS-BMD, and Heel BMD, respectively. We did not find any evidence of a causal effect (*p* > 0.05) of BMD at different sites on AD by IVW analysis (Figure 6; Supplementary Figure 7), and similar results were obtained by the MR–Egger method, weighted median method, and weighted mode method (Figure 6). Cochran’s Q test showed no heterogeneity except for Heel–BMD (*p* < 0.05). All tests for Egger’s regression were negative (*p* > 0.05; Supplementary Table 3; Supplementary Figures 8A-E), indicating that our MR results were not influenced by horizontal pleiotropy. Because of the heterogeneity of Heel-BMD, the analysis was performed using weighted medians (Nazarzadeh et al., 2020), and the results still showed that there was no causal relationship of BMD at different sites on AD (Figure 6). The leave-one-out sensitivity test results indicated that no individual SNP had a potential influence on the final results (Supplementary Figures 8F–J).

### Effects of BMD at different ages on AD

**Figure 7:**
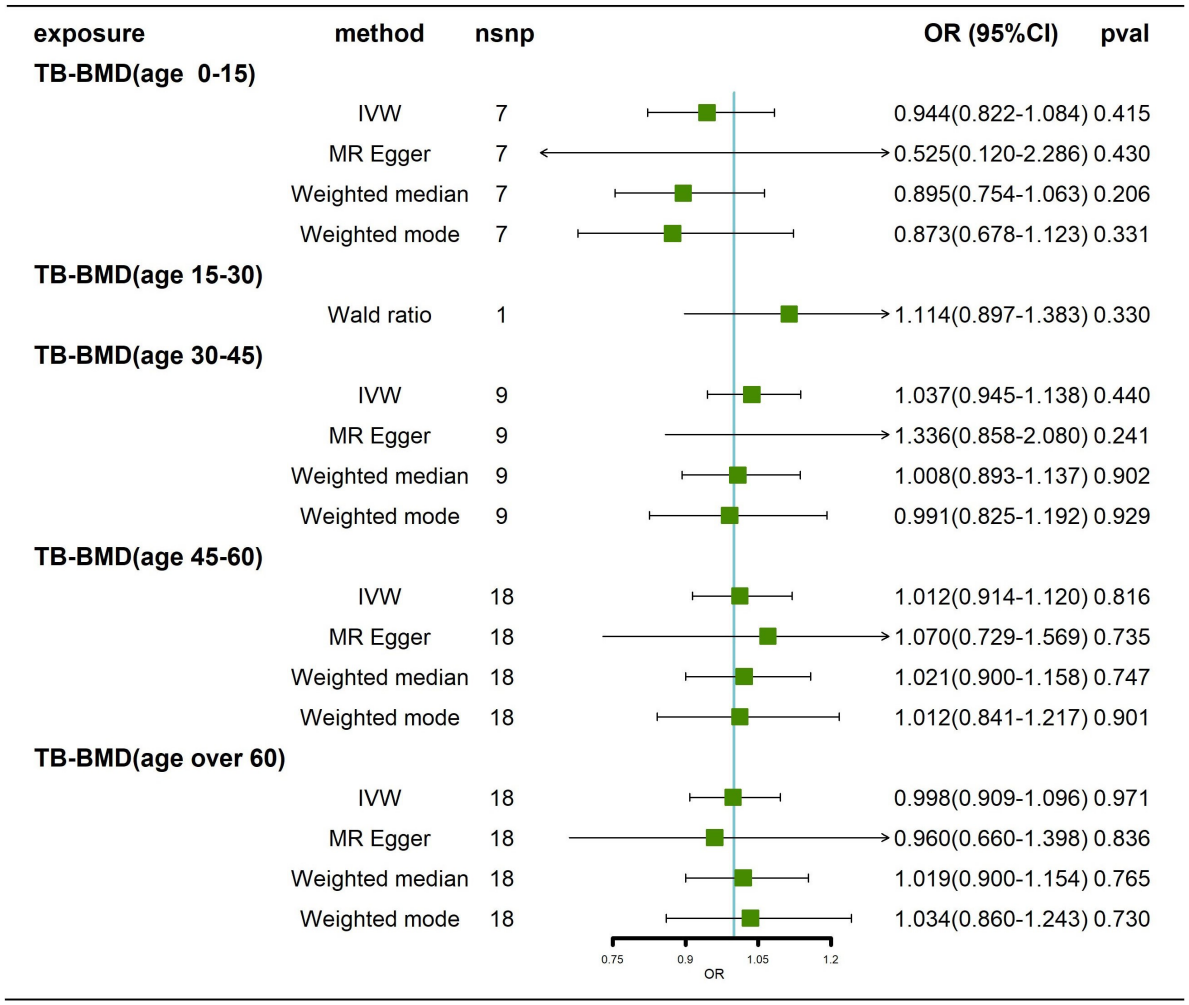
Causal effects of BMD in different age groups on AD. AD: Alzheimer’s disease; TB-BMD: total body bone mineral density; SNP, single nucleotide polymorphism; IVW, inverse variance weighted; nsnp, number of single nucleotide polymorphisms; CI, confidence interval.

We obtained 7, 1, 9, 18 and 18 SNPs from GWASs for TB-BMD in subjects aged 0–15 years, 15–30 years, 30–45 years, 45–60 years, and over 60 years, respectively. In general, there was no causal correlation of BMD at different ages with AD, regardless of whether the IVW method or MR–Egger method was used (Figure 7; Supplementary Figure 9). As only 1 SNP was found for BMD in the age range of 15–30 years, heterogeneity and horizontal pleiotropy tests were not needed. All tests for Cochran’s Q tests and Egger’s regression tests for AD were negative (*p* > 0.05) for BMD in the remaining age groups (Supplementary Table 4; Supplementary Figures 10A-D), indicating that MR results were not affected by heterogeneity or horizontal pleiotropy. Finally, the results of the leave-one-out sensitivity test showed no potential influence of any individual SNP on causality (Supplementary Figures 10E–H). Ultimately, we found that there was no significant causal effect of BMD at different ages on AD.

## Discussion

In this study, we used bidirectional MR to test for a causal relationship in either direction between osteoporosis and AD. Although we used the largest publicly available GWAS dataset for analysis and stratified BMD by age and site, there was no evidence of a genetic causal relationship in either direction between osteoporosis and AD. Therefore, based on the results of our MR analysis, there is no evidence of any causal relationship between osteoporosis and AD.

Although we found no causal link between osteoporosis and AD, previous observational studies have shown a strong association between osteoporosis and AD. A cohort study found a 1.49-fold higher prevalence of AD in the osteoporosis group than in the control group after correcting for age and sex, and its results suggested that the presence of osteoporosis increases the potential for AD in adults over 40 years old (Kwon et al., 2021). Another cohort study also found that osteoporosis was positively associated with cognitive and functional decline and that subjects with BMD values in the lowest quartile had a 2-fold increased risk of AD transition compared to controls (Zhou et al., 2014). This may be related to the occurrence of risk genes (Aβ42 and amyloid precursor peptide) for AD that also predispose patients to osteoporosis (Xia et al., 2013; Li et al., 2014). Based on the results of MR, this study found no genetic causality between osteoporosis and AD, and we suggest that the association observed in the clinic may be due to a similar pathogenesis.

Previous studies have found a possible common pathogenesis of osteoporosis and AD. Osteoclasts and microglia are involved in the pathogenesis of osteoporosis and AD, respectively (Ulland and Colonna, 2018; Tsukasaki and Takayanagi, 2019). Key signals and pathways shared between osteoclasts and microglia, namely, myeloid cells 2 (TREM2) /DNAX Adaptor Protein 12 kD (TREM2/DAP12), macrophage colony-stimulating factor (M-CSF, also known as CSF1) and C-C-Motif Chemokine Receptor 5 (CCR5), converge through the *Pyk2* pathway, which may be a common pathway for genetic correlation between osteoporosis and AD (Lee et al., 2021). Other studies have noted that osteoporosis and AD exhibit reduced glucose metabolism in the bone and brain prior to disease onset and that the cellular energy supply is compromised through the impaired effects of AKT on glucose uptake (Fehsel and Christl, 2022). Aberrant Wnt/β-catenin signaling is also a common mechanism affecting osteoporosis and AD (Li et al., 2013; Riise et al., 2015; Folke et al., 2019). Dengler-Crish et al. (2018) found that htau mice (an AD mouse model) had significantly lower BMD than C57BL/6 J mice, and htau mice exhibited inhibition of the Wnt/β-catenin signaling pathway in both the bone and brain. Guo et al. (2016) suggested that Dickkopf-related protein 1 (DKK1), a key endogenous antagonist of the Wnt signaling pathway, may be a common risk molecule for AD and osteoporosis. Recent studies have proposed new mechanisms for the correlation between osteoporosis and AD (Jiang et al., 2022). The results revealed that young osteocyte-derived extracellular vesicles (OCY-EVs) could access the brain to improve cognitive function in AD mice, and that inhibiting the secretion of OCY-EVs increased cognitive impairment in AD mice, revealing a “bone-brain axis” information signaling mechanism. This provides possible reasons why, although our results show no causal relationship between osteoporosis and AD, an association between osteoporosis and AD is observed in the clinic: this association may be driven by a common pathogenesis or metabolic interaction.

There exists a common pathogenesis between osteoporosis and AD; therefore, it is worthwhile to explore whether the treatment for each disease affects the other. Recently, it was found that osteoblasts express specific acetylcholine receptors (AChRs) and cholinergic components, and inhibition of AChRs appears to decrease bone turnover (Inkson et al., 2004; Sato et al., 2010). Acetylcholinesterase inhibitors (AChEIs), a group of drugs that stimulate the AChRs by inhibiting the action of acetylcholinesterase and increasing the level of intrasynaptic acetylcholine, are now widely used in the treatment of AD (Pepeu and Giovannini, 2009). A 5-year retrospective case–control study found that among patients with AD, those using AChEIs had a lower risk of hip fracture than those not using (Tamimi et al., 2012), and a larger case–control study supported this conclusion (Tamimi et al., 2018). Tamimi et al. (2018) noted that past use of AChEIs provided no protective effect against osteoporotic fractures, possibly because when the AChEIs use was interrupted, the bone protective effect disappeared. In addition, the inverse relationship between adherence to AChEIs and the risk of osteoporotic fractures suggests a protective effect on bone (Tamimi et al., 2018). Another retrospective cohort study also found an increase in hip fracture healing and bone quality with the use of AChEIs in patients with AD (Eimar et al., 2013). A recent case–control study involving 9,470 patients had contrasting results, with its finding that the use of AChEIs, increased the risk of osteoporotic fractures, but there were significant differences between the two groups at baseline, such as smoking, comorbidities, and comorbidities, which may have influences the results of the study (Won et al., 2020). As to whether treatment of osteoporosis affects AD, Zameer et al. (2018) noted a reduced propensity to develop dementia in patients with osteoporosis treated with bisphosphonates. Another retrospective cohort study also found that patients with osteoporosis treated with bisphosphonates had a significantly reduced risk of AD, which also implies that treatment of osteoporosis with bisphosphonates may reduce the incidence of AD (Chang et al., 2014). However, large randomized controlled trials are still needed to further investigate whether anti-osteoporosis treatment improves AD and/or AD treatment mitigates the degree of osteoporosis in clinical practice; this is one of the directions for future research (Hadj Sadok and de Oliveira, 2019).

To the best of our knowledge, there are no reported MR studies on the effects of osteoporosis on AD and vice versa. Our study used several IVs from large GWAS of AD and BMD to increase the statistical power to detect causality, allowing for more precise assessment of effect sizes. In addition, our stratified analysis of BMD by age and site clarified the causal association between AD and BMD at different ages and sites.

However, there are some limitations of our study. First, we did not perform a stratified analysis of the causal effect of gender on the association between osteoporosis and AD. Second, the study population included in this MR analysis was of European ancestry. Whether this result can be replicated in Asian populations remains to be explored.

In conclusion, our findings suggest that there is no causal relationship in either direction between osteoporosis and AD. According to our findings, although there is no causal relationship between them, they share similar pathogenesis and pathways. It is reasonable to routinely prevent osteoporosis in patients with AD, and vice versa. Proper management of AD and osteoporosis is essential to reduce the risk of developing both. Future multidisciplinary cooperation may play a very important role in clinical practice and influence the prognosis of these disease.

## Data availability statement

Publicly available datasets were analyzed in this study. This data can be found at: All GWAS summary statistics can be downloaded from open GWAS for exposures (https://gwas.mrcieu.ac.uk/), GWAS catalog (https://www.ebi.ac.uk/gwas/ and https://data.bris.ac.uk/).

## Author contributions

HH performed the study and wrote the manuscript. JM performed the main data analysis. YC contributed to the data analysis and manuscript revision. HD and SN revised the manuscript. WZ and XF designed the study. All authors contributed to the article and approved the submitted version.

## Funding

This work was supported by the Scientific Research Project from the Education Department of Fujian Province (JAT210403), Foreign Cooperation Project of Science and Technology, Fujian Province (2021I0012), The National Science Foundation Grant of China (82072458, 82171370), Joint Funds for the Innovation of Science and Technology, Fujian Province, China (2019Y9136, 2021Y9125), Fujian Medical Innovation Grant, China (2020CXA038), Fujian Orthopaedic Bone and Joint Disease and Sports Rehabilitation Clinical Medical Research Center (2020Y2002), and Natural Science Foundation of Fujian Province (2022 J011432).

## Conflict of interest

The authors declare that the research was conducted in the absence of any commercial or financial relationships that could be construed as a potential conflict of interest.

## Publisher’s note

All claims expressed in this article are solely those of the authors and do not necessarily represent those of their affiliated organizations, or those of the publisher, the editors and the reviewers. Any product that may be evaluated in this article, or claim that may be made by its manufacturer, is not guaranteed or endorsed by the publisher.
