# Supplementary figures and images for: No genetic causal association between Alzheimer’s disease and osteoporosis: A bidirectional two-sample Mendelian randomization study
Source: Front Aging Neurosci. 2023 Jan 25;15:1090223. doi: 10.3389/fnagi.2023.1090223 (PMC9905740; doi:10.3389/fnagi.2023.1090223)

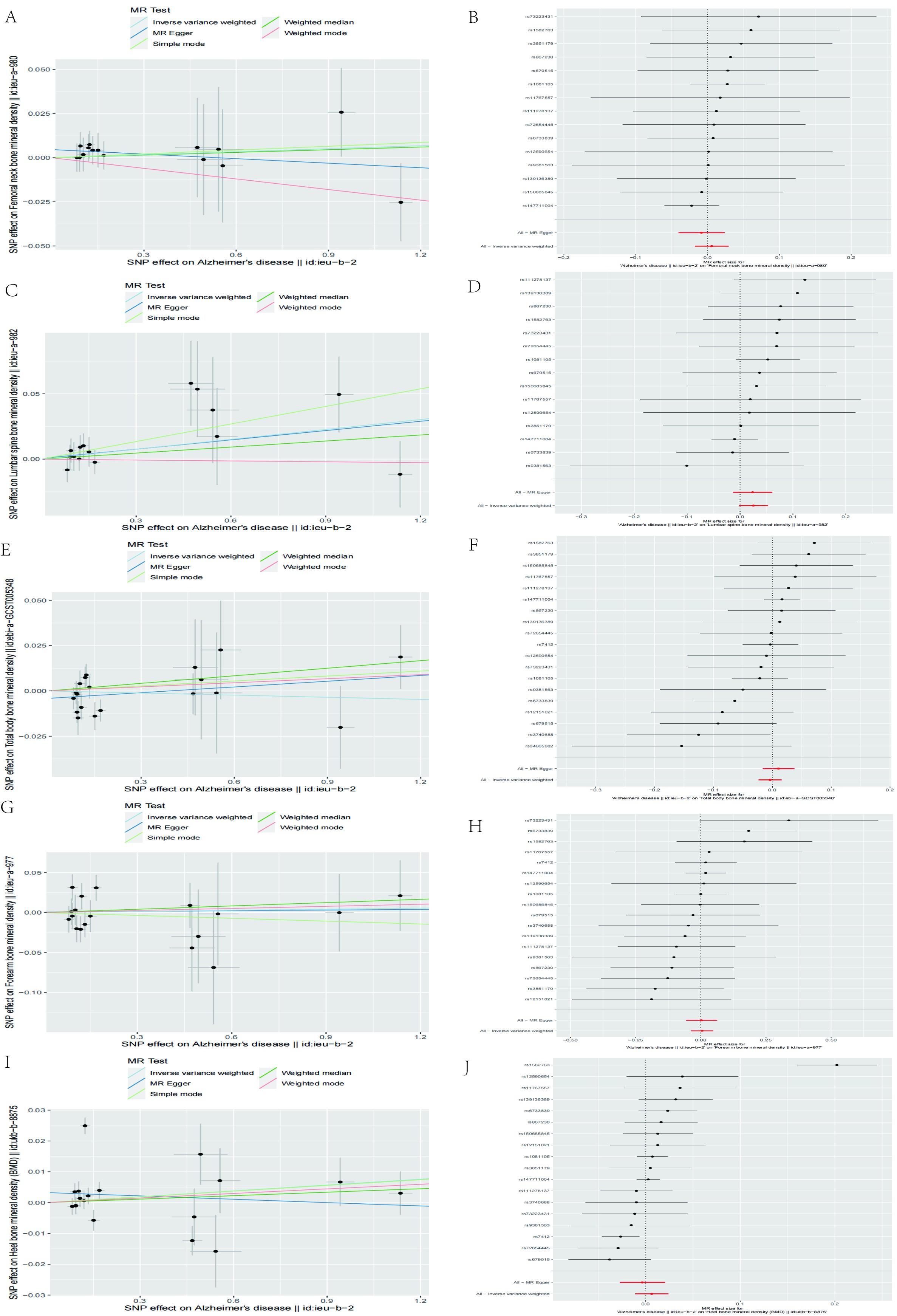

Supplement: Supplementary file 1 [file Image_1.JPEG]

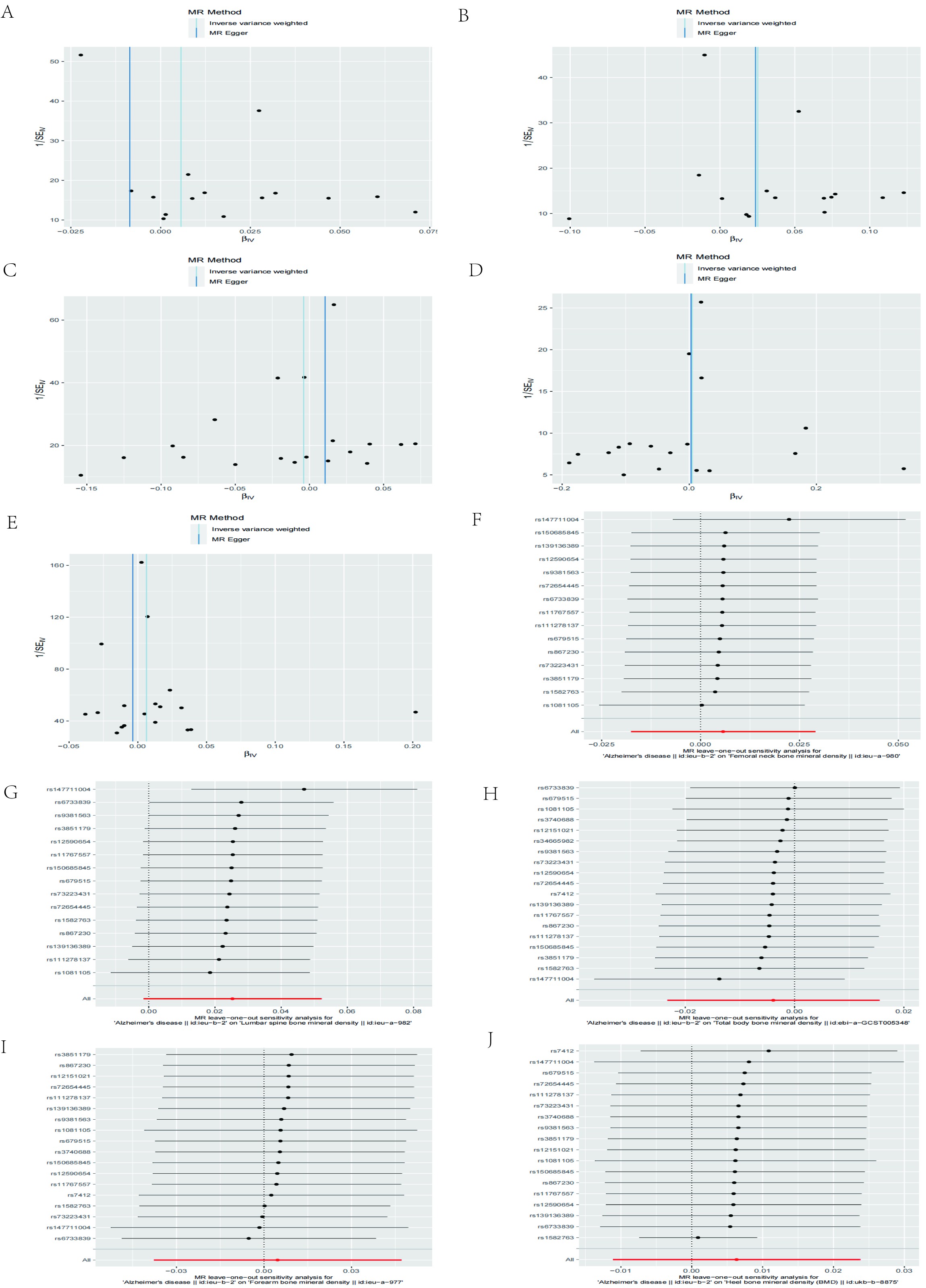

Supplement: Supplementary file 2 [file Image_2.JPEG]

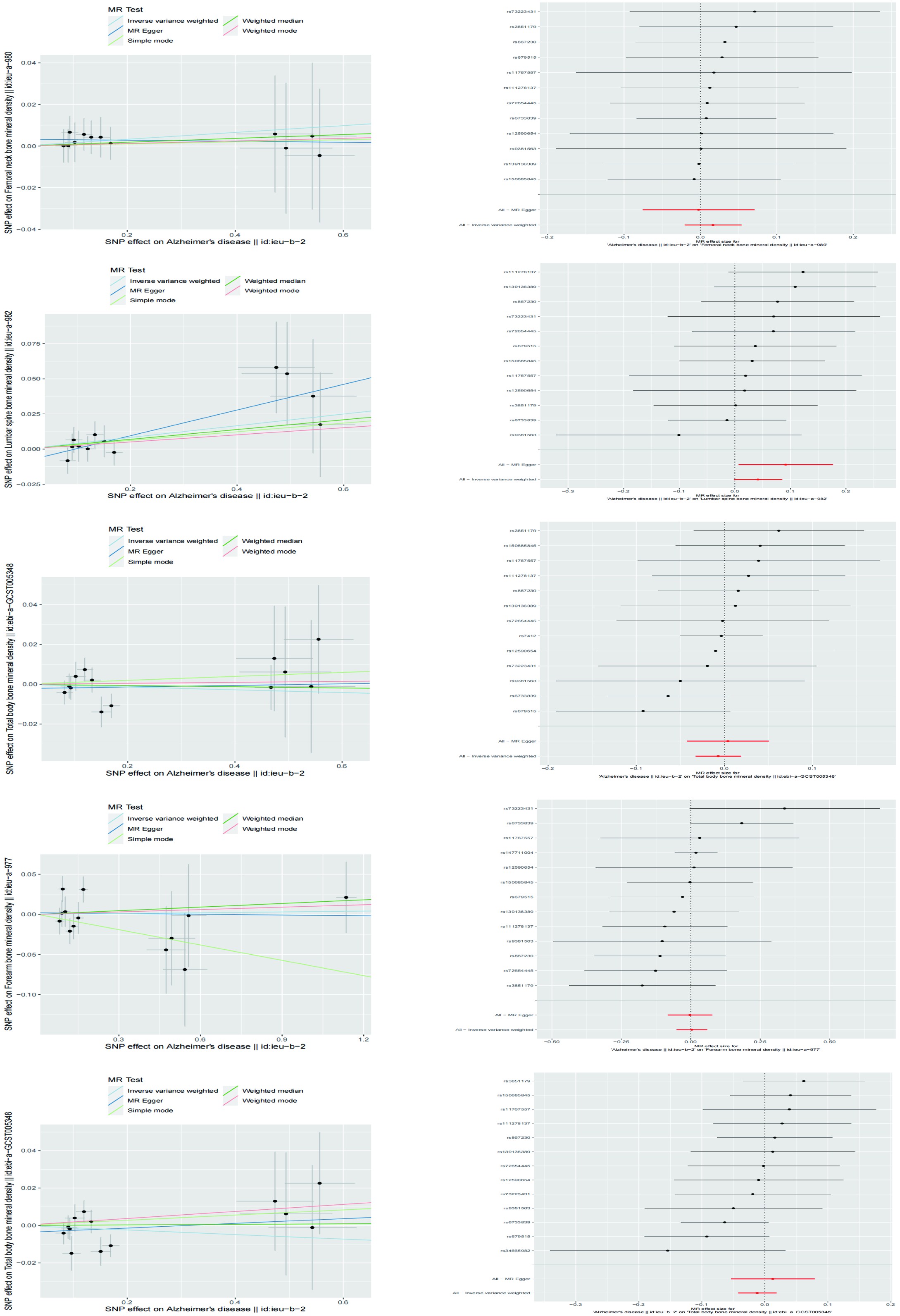

Supplement: Supplementary file 3 [file Image_3.JPEG]

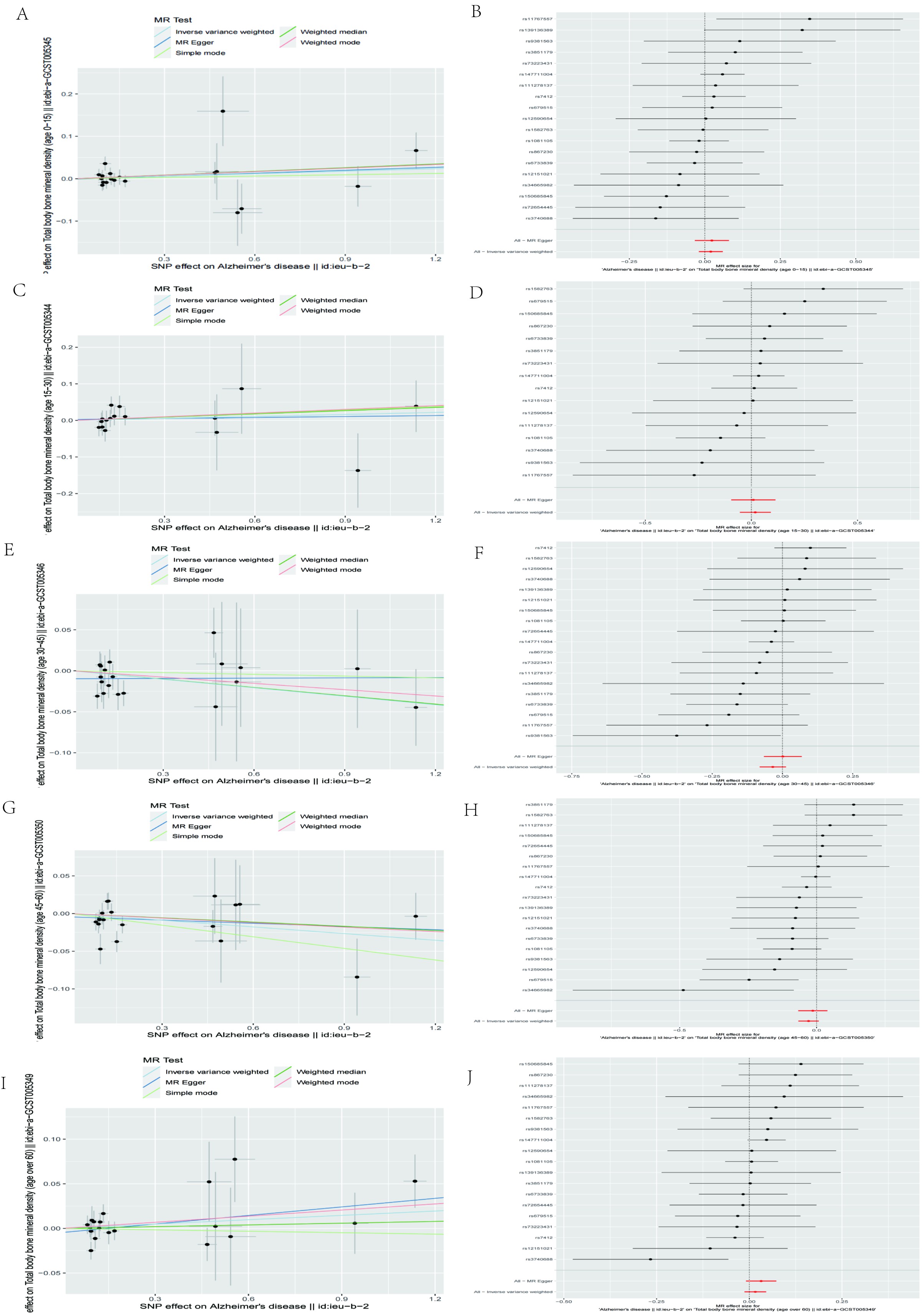

Supplement: Supplementary file 4 [file Image_4.JPEG]

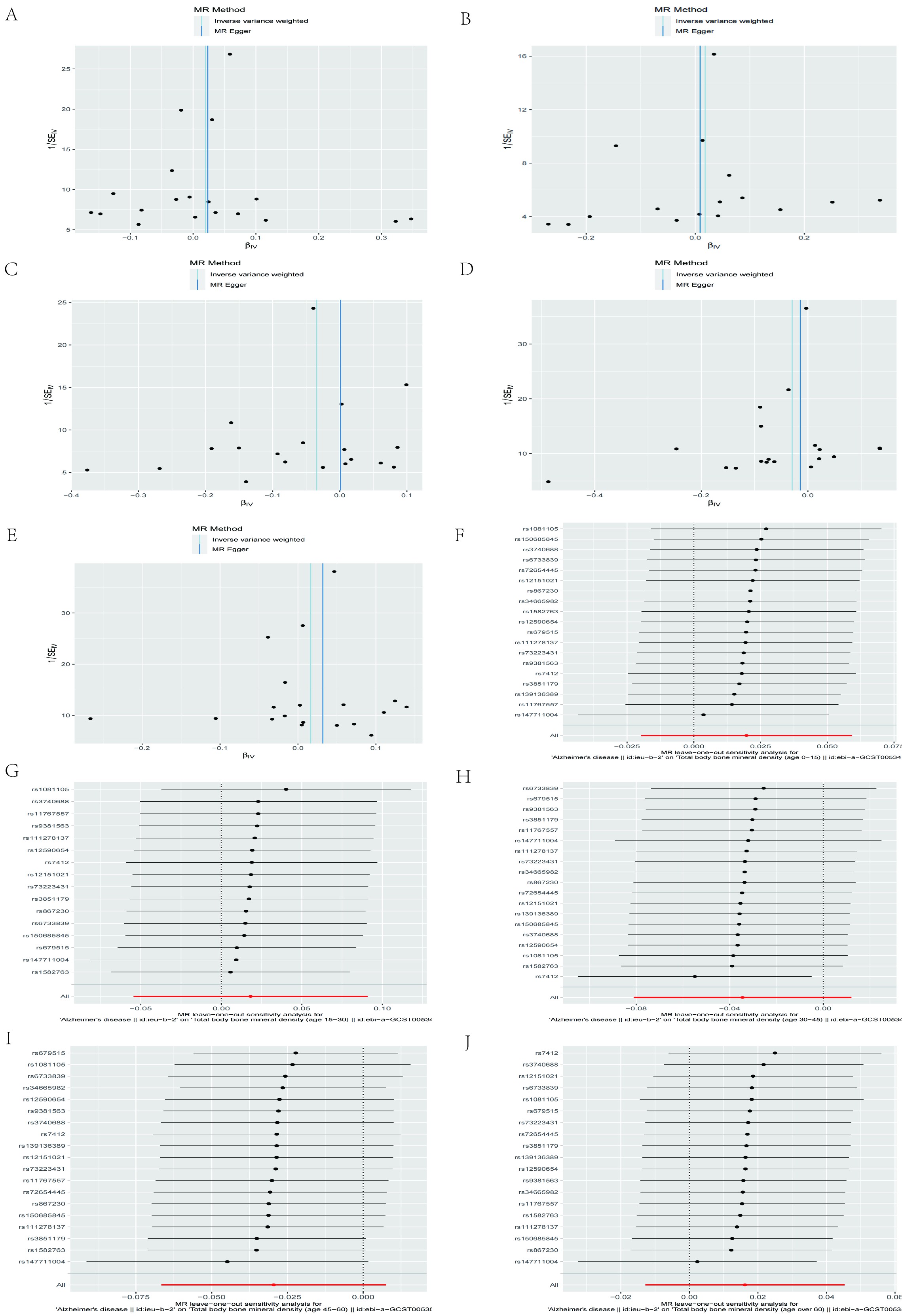

Supplement: Supplementary file 5 [file Image_5.JPEG]

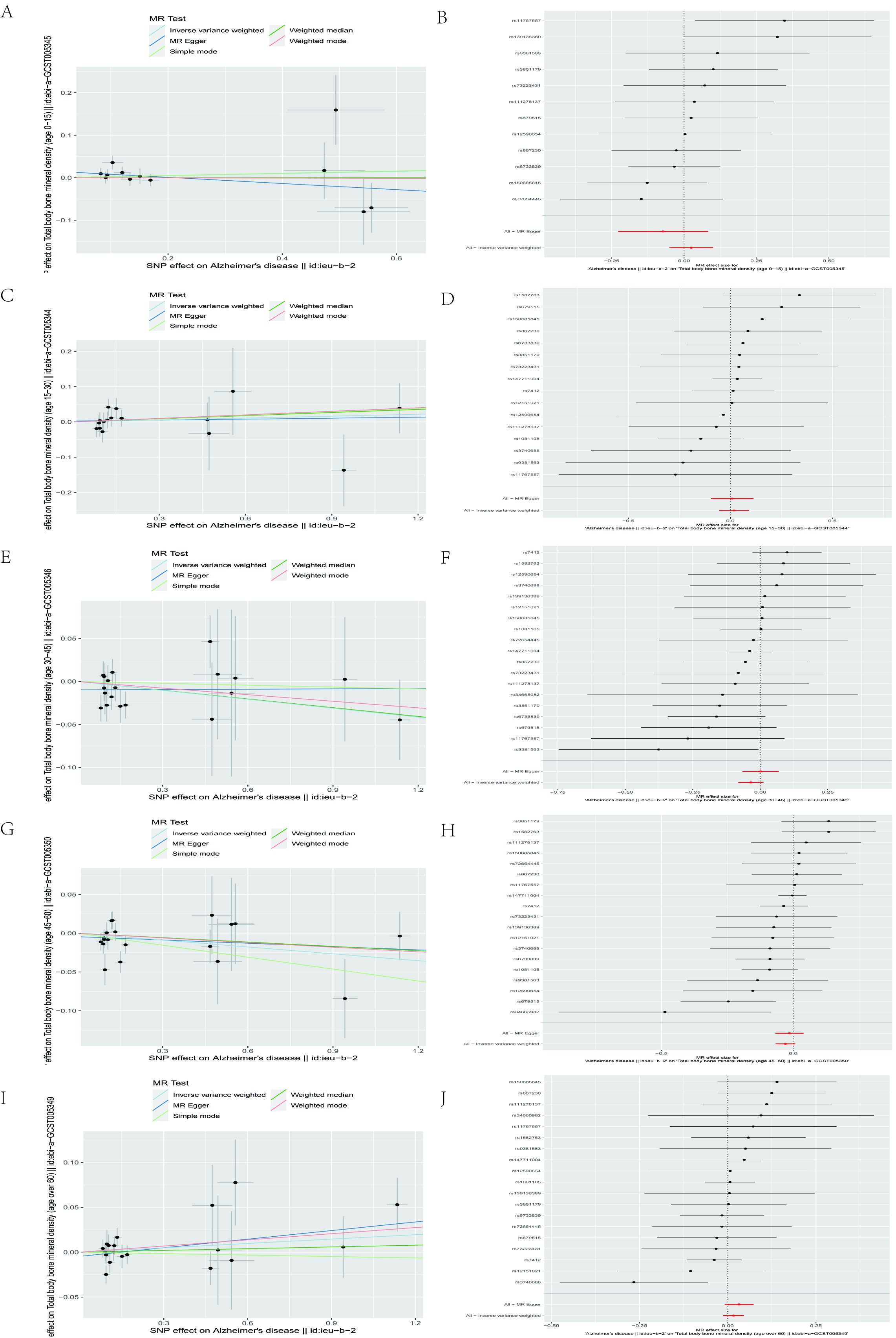

Supplement: Supplementary file 6 [file Image_6.JPEG]

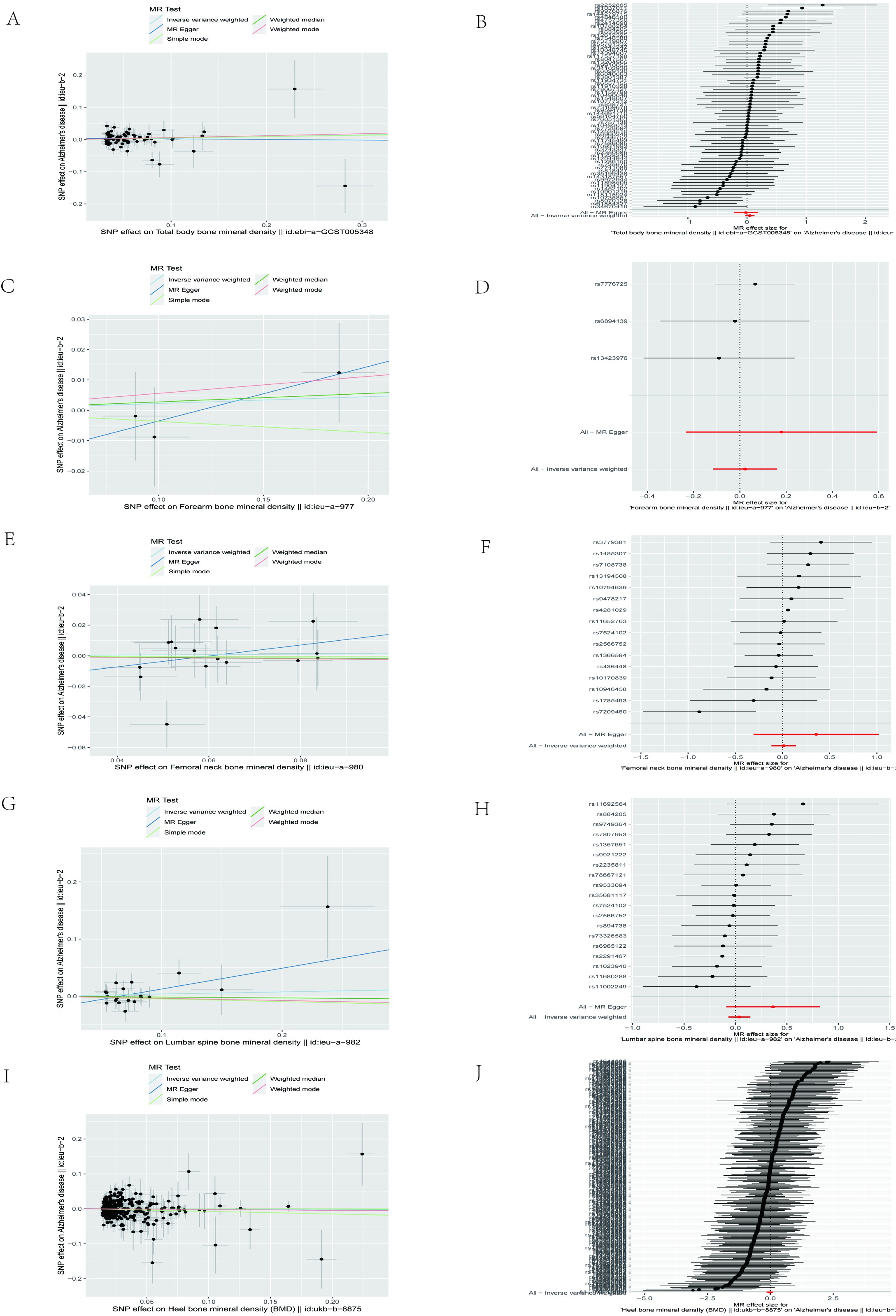

Supplement: Supplementary file 7 [file Image_7.JPEG]

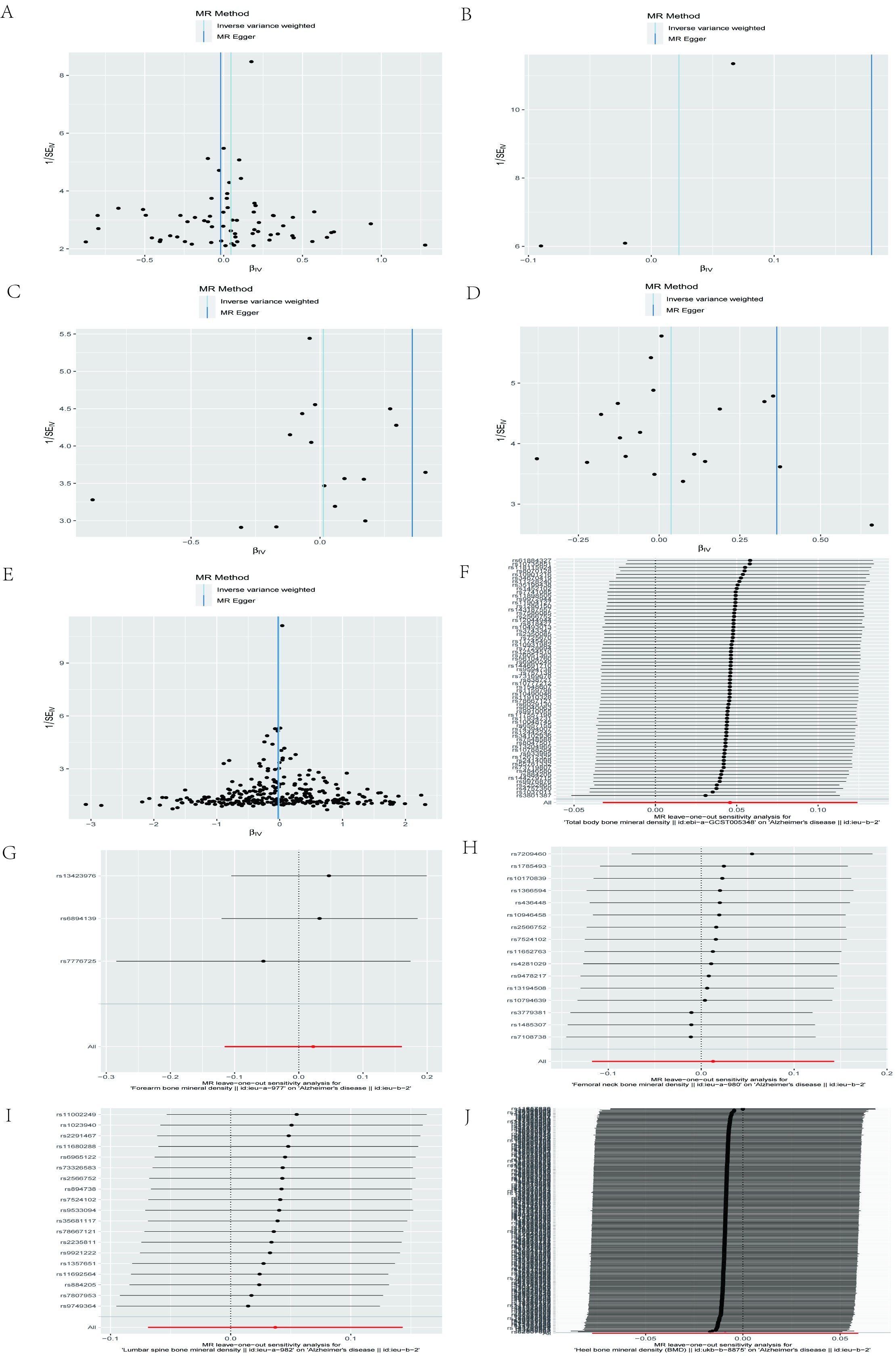

Supplement: Supplementary file 8 [file Image_8.JPEG]

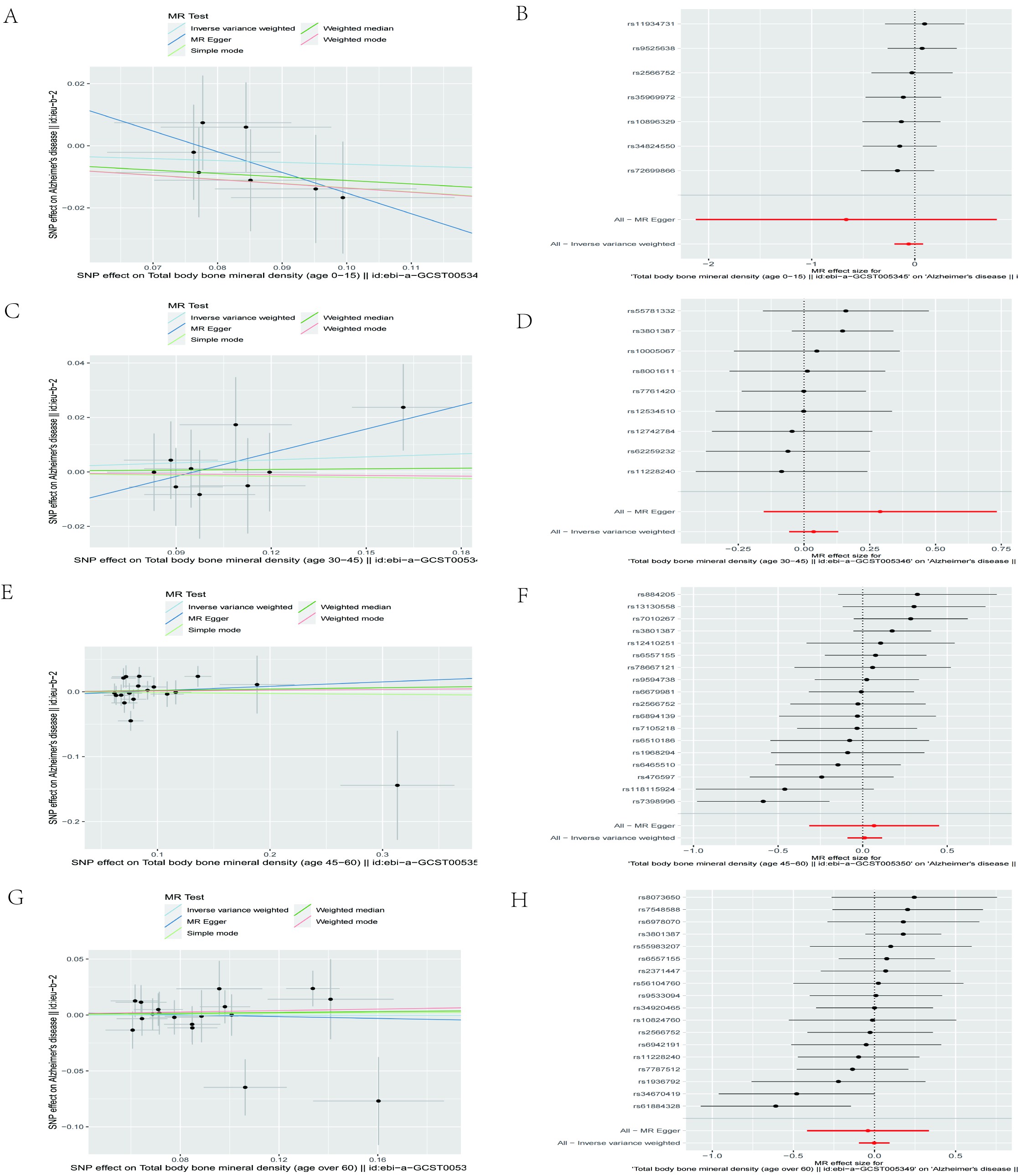

Supplement: Supplementary file 9 [file Image_9.JPEG]

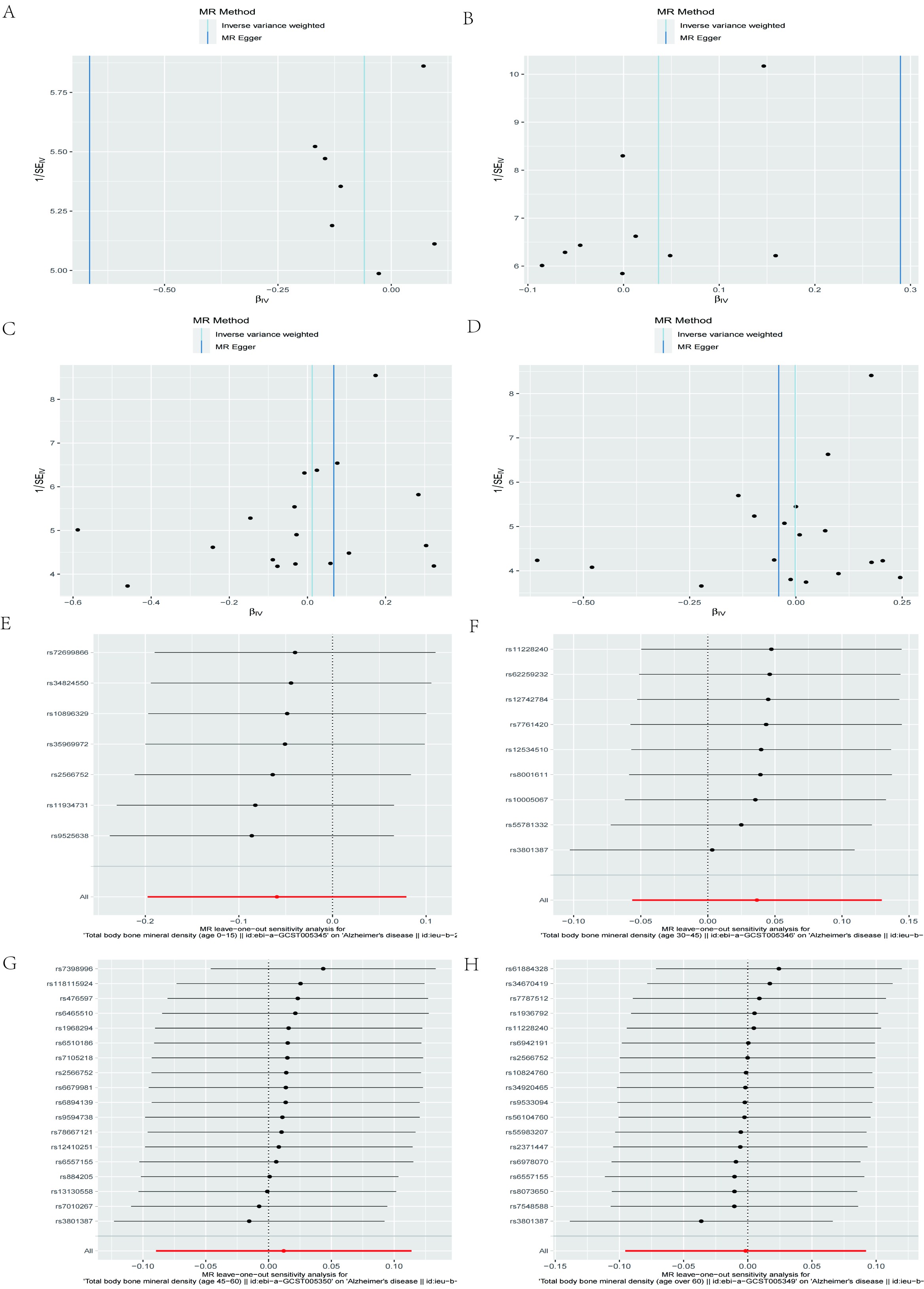

Supplement: Supplementary file 10 [file Image_10.JPEG]
