# Supplementary material for: No genetic causal association between Alzheimer’s disease and osteoporosis: A bidirectional two-sample Mendelian randomization study
Source: Front Aging Neurosci. 2023 Jan 25;15:1090223. doi: 10.3389/fnagi.2023.1090223 (PMC9905740; doi:10.3389/fnagi.2023.1090223)
Supplement: Supplementary file 11 [file Table_1.DOCX]

**Supplementary Table 1. The Heterogeneity tests and Directional horizontal pleiotropy test for AD on BMD at different sites**

| **Outcome** | **Methods** | **Cochran’s Q (P-value)** | **MR-Egger intercept (P-value)** |
| --- | --- | --- | --- |
| FN-BMD | MR Egger | 3.2917 (0.997) | 0.0047 (0.221) |
| FN-BMD | Inverse variance weighted | 4.9411 (0.987) |  |
| LS-BMD | MR Egger | 10.0841 (0.687) | 0.0005 (0.907) |
| LS-BMD | Inverse variance weighted | 10.0982 (0.755) |  |
| TB-BMD | MR Egger | 20.0115 (0.274) | -0.0041 (0.153) |
| TB-BMD | Inverse variance weighted | 22.6444 (0.205) |  |
| FA-BMD | MR Egger | 15.7196 (0.473) | 0.0006 (0.930) |
| FA-BMD | Inverse variance weighted | 15.7275 (0.543) |  |
| Heel BMD | MR Egger | 100.0997 (3.318e-14) | 0.0033 (0.252) |
| Heel BMD | Inverse variance weighted | 108.9395 (1.910e-15) |  |
