# Supplementary material for: No genetic causal association between Alzheimer’s disease and osteoporosis: A bidirectional two-sample Mendelian randomization study
Source: Front Aging Neurosci. 2023 Jan 25;15:1090223. doi: 10.3389/fnagi.2023.1090223 (PMC9905740; doi:10.3389/fnagi.2023.1090223)
Supplement: Supplementary file 12 [file Table_2.DOCX]

**Supplementary Table 2. The Heterogeneity tests and Directional horizontal pleiotropy test for AD on BMD at different age groups**

| **Outcome** | Methods | Cochran’sQ (P-value) | MR-Egger intercept (P-value) |
| --- | --- | --- | --- |
| TB-BMD (0-15 years) | MR Egger | 16.9621 (0.457) | -0.0010 (0.862) |
| TB-BMD (0-15 years) | Inverse variance weighted | 16.9932 (0.524) |  |
| TB-BMD (15-30 years) | MR Egger | 9.7792 (0.778) | 0.0025 (0.812) |
| TB-BMD (15-30 years) | Inverse variance weighted | 9.8383 (0.830) |  |
| TB-BMD (30-45 years) | MR Egger | 13.9411 (0.671) | -0.0097 (0.167) |
| TB-BMD (30-45 years) | Inverse variance weighted | 16.0248 (0.591) |  |
| TB-BMD (45-60 years) | MR Egger | 22.5486 (0.165) | -0.0044 (0.435) |
| TB-BMD (45-60 years) | Inverse variance weighted | 23.3981 (0.176) |  |
| TB-BMD (over 60 years) | MR Egger | 17.1480 (0.444) | -0.0046 (0.299) |
| TB-BMD (over 60 years) | Inverse variance weighted | 18.3072 (0.436) |  |
