# Supplementary material for: No genetic causal association between Alzheimer’s disease and osteoporosis: A bidirectional two-sample Mendelian randomization study
Source: Front Aging Neurosci. 2023 Jan 25;15:1090223. doi: 10.3389/fnagi.2023.1090223 (PMC9905740; doi:10.3389/fnagi.2023.1090223)
Supplement: Supplementary file 13 [file Table_3.DOCX]

**Supplementary Table 3. The Heterogeneity tests and Directional horizontal pleiotropy test for BMD at different sites on AD.**

| **Exposure** | **Methods** | **Cochran’sQ (P-value)** | **MR-Egger intercept (P-value)** |
| --- | --- | --- | --- |
| FN-BMD | MR Egger | 14.7282 (0.397) | -0.0216 (0.317) |
| FN-BMD | Inverse variance weighted | 15.8600 (0.391) |  |
| LS-BMD | MR Egger | 12.9531 (0.740) | -0.0240 (0.165) |
| LS-BMD | Inverse variance weighted | 15.0603 (0.658) |  |
| TB-BMD | MR Egger | 73.3214 (0.251) | 0.0038 (0.503) |
| TB-BMD | Inverse variance weighted | 73.8246 (0.265) |  |
| FA-BMD | MR Egger | 0.1528 (0.696) | -0.0214 (0.574) |
| FA-BMD | Inverse variance weighted | 0.7780 (0.678) |  |
| Heel BMD | MR Egger | 382.1676 (0.0004) | 0.0006 (0.763) |
| Heel BMD | Inverse variance weighted | 382.2863 (0.0005) |  |
