# Supplementary material for: No genetic causal association between Alzheimer’s disease and osteoporosis: A bidirectional two-sample Mendelian randomization study
Source: Front Aging Neurosci. 2023 Jan 25;15:1090223. doi: 10.3389/fnagi.2023.1090223 (PMC9905740; doi:10.3389/fnagi.2023.1090223)
Supplement: Supplementary file 14 [file Table_4.DOCX]

**Supplementary Table 4. The Heterogeneity tests and Directional horizontal pleiotropy test for BMD at different age groups on AD.**

| **Exposure** | **Methods** | **Cochran’s Q (P-value)** | **MR-Egger intercept (P-value)** |
| --- | --- | --- | --- |
| TB-BMD (age 0-15 years) | MR Egger | 1.3566 (0.929) | 0.0497 (0.468) |
| TB-BMD (age 0-15 years) | Inverse variance weighted | 1.9739 (0.922) |  |
| TB-BMD (age 30-45 years) | MR Egger | 1.8869 (0.966) | -0.0277 (0.290) |
| TB-BMD (age 30-45 years) | Inverse variance weighted | 3.1979 (0.921) |  |
| TB-BMD (age 45-60 years) | MR Egger | 22.9514 (0.115) | -0.0050 (0.772) |
| TB-BMD (age 45-60 years) | Inverse variance weighted | 23.0759 (0.147) |  |
| TB-BMD (age over 60 years) | MR Egger | 16.9550 (0.389) | 0.0034 (0.837) |
| TB-BMD (age over 60 years) | Inverse variance weighted | 17.0013 (0.454) |  |
