# Supplementary material for: No genetic causal association between Alzheimer’s disease and osteoporosis: A bidirectional two-sample Mendelian randomization study
Source: Front Aging Neurosci. 2023 Jan 25;15:1090223. doi: 10.3389/fnagi.2023.1090223 (PMC9905740; doi:10.3389/fnagi.2023.1090223)
Supplement: Supplementary file 15 [file Data_Sheet_1.docx]

**Supplementary Figure 1**: Scatter plots of Mendelian randomization (MR) estimates of the causal relationship of AD on BMD at different sites. (A) FN-BMD, (C) LS-BMD, (E) TB-BMD, (G) FA-BMD, and (I) Heel BMD. The slopes of the line represent the causal effect of each method. Forest plots of the causal effects of AD-associated SNPs on BMD at different sites. (B) FN-BMD, (D) LS-BMD, (F) TB-BMD, (H) FA-BMD, and (J) Heel-BMD. AD: Alzheimer's disease; FN-BMD: femoral neck bone mineral density; LS-BMD: lumbar spine bone mineral density; TB-BMD: total body bone mineral density; FA-BMD: forearm bone mineral density; Heel-BMD: heel bone mineral density; SNP, single nucleotide polymorphism; IVW, inverse variance weighted.

**Supplementary Figure 2:** (A-E) Funnel plots of the effect of AD on BMD at different sites. (A) FN-BMD, (B) LS-BMD, (C) TB-BMD, (D) FA-BMD, and (E) Heel-BMD. (F-J) Leave-one-out analysis plots for the effect of AD on BMD at different sites. (F) FN-BMD , (G) LS-BMD, (H) TB-BMD, (I) FA-BMD, and (J) Heel-BMD. AD: Alzheimer's disease; FN-BMD: femoral neck bone mineral density; LS-BMD: lumbar spine bone mineral density; TB-BMD: total body bone mineral density; FA-BMD: forearm bone mineral density; Heel-BMD: heel bone mineral density.

**Supplementary Figure 3:** Scatter plots of Mendelian randomization estimates of the causal effect of AD on BMD at different sites after removal of potentially pleiotropic SNPs. (A) FN-BMD, (C) LS-BMD, (E) TB-BMD, (G) FA-BMD, and (I) Heel-BMD. The slopes of the line represents the causal effect of each method. Forest plot of the causal effects of AD-associated SNPs on BMD at different sites after removal of potentially pleiotropic SNPs. (B) FN-BMD, (D) LS-BMD, (F) TB-BMD, (H) FA-BMD, and (J) Heel BMD. AD: Alzheimer's disease; FN-BMD: femoral neck bone mineral density; LS-BMD: lumbar spine bone mineral density; TB-BMD: total body bone mineral density; FA-BMD: forearm bone mineral density; Heel-BMD: heel bone mineral density; SNP, single nucleotide polymorphism; IVW, inverse variance weighted.

**Supplementary Figure 4:** Scatter plots of Mendelian randomization (MR) estimates of the causal relationship of AD on BMD in different age groups. (A) TB-BMD (age 0-15 years), (C) TB-BMD (age 15-30 years), (E) TB-BMD (age 30-45 years), (G) TB-BMD (age 45-60 years), and (I) TB-BMD (age over 60 years). The slopes of the line represent the causal effect of each method. Forest plots of the causal effects of AD-associated SNPs on BMD in different age groups after removal of potentially pleiotropic SNPs. (B) TB-BMD (age 0-15 years), (D) TB-BMD (age 15-30 years), (F) TB-BMD (age 30-45 years), (H) TB-BMD (age 45-60 years), and (J) TB-BMD (age over 60 years). AD: Alzheimer's disease; TB-BMD: total body bone mineral density; SNP, single nucleotide polymorphism; IVW, inverse variance weighted.

**Supplementary Figure 5:** (A-E) Funnel plots of the causal effect of AD on BMD in different age groups. (A) TB-BMD (age 0-15 years), (B) TB-BMD (age 15-30 years), (C) TB-BMD (age 30-45 years), (D) TB-BMD (age 45-60 years), and (E) TB-BMD (age over 60 years). (F-J) Leave-one-out analysis plots for the causal effect of AD on BMD at different age groups. (F) TB-BMD (age 0-15 years), (G) TB-BMD (age 15-30 years), (H) TB-BMD (age 30-45 years), (I) TB-BMD (age 45-60 years), and (J) TB-BMD (age over 60 years). AD: Alzheimer's disease; TB-BMD: total body bone mineral density; SNP, single nucleotide polymorphism; IVW, inverse variance weighted.

**Supplementary Figure 6**: Scatter plots of Mendelian randomization estimates of the causal relationship of AD on BMD at different sites after removal of potentially pleiotropic SNPs. (A) TB-BMD (age 0-15 years), (C) TB-BMD (age 15-30 years), (E) TB-BMD (age 30-45 years), (G) TB-BMD (age 45-60 years), and (I) TB-BMD (age over 60 years). The slopes of the line represent the causal effect of each method. Forest plot of the causal effects of AD-associated SNPs on BMD at different sites after removal of potentially pleiotropic SNPs. (B) TB-BMD (age 0-15 years), (D) TB-BMD (age 15-30 years), (F) TB-BMD (age 30-45 years), (H) TB-BMD (age 45-60 years), and (J) TB-BMD (age over 60 years). AD: Alzheimer's disease; TB-BMD: total body bone mineral density; SNP, single nucleotide polymorphism; IVW, inverse variance weighted.

**Supplementary Figure 7:** Scatter plots of Mendelian randomization estimates of the causal relationship of BMD at different sites with AD. (A) TB-BMD, (C) FA-BMD, (E) FN-BMD, (G) LS-BMD, and (I) Heel-BMD. The slopes of the line represent the causal effect of each method. Forest plots of the causal effects of site-specific BMD-associated SNPs on AD. (B) TB-BMD, (D) FA-BMD, (F) FN-BMD, (H) LS-BMD, and (J) Heel-BMD. AD: Alzheimer's disease; FN-BMD: femoral neck bone mineral density; LS-BMD: lumbar spine bone mineral density; TB-BMD: total body bone mineral density; FA-BMD: forearm bone mineral density; Heel-BMD: heel bone mineral density; SNP, single nucleotide polymorphism; IVW, inverse variance weighted.

**Supplementary Figure 8**: (A-E) Funnel plots of the causal effect of BMD at different sites on AD. (A) TB-BMD, (B) FA-BMD, (C) FN-BMD, (D) LS-BMD, and (E) Heel-BMD. (F-J) Leave-one-out analysis plots for the causal effect of BMD at different sites on AD. (F) TB-BMD, (G) FA-BMD, (H) FN-BMD, (I) LS-BMD, and (J) Heel-BMD. AD: Alzheimer's disease; FN-BMD: femoral neck bone mineral density; LS-BMD: lumbar spine bone mineral density; TB-BMD: total body bone mineral density; FA-BMD: forearm bone mineral density; Heel-BMD: heel bone mineral density.

**Supplementary Figure 9:** Scatter plots of Mendelian randomization estimates of the causal effect of BMD in different age groups on AD. (A) TB-BMD (age 0-15 years), (C) TB-BMD (age 30-45 years), (E) TB-BMD (age 45-60 years), and (G) TB-BMD (age over 60 years). The slopes of the line represents the causal effect of each method. Forest plots of the causal effects of age-specific BMD-associated SNPs on AD. (B) TB-BMD (age 0-15 years), (D) TB-BMD (age 30-45 years), (F) TB-BMD (age 45-60 years), and (H) TB-BMD (age over 60 years). AD: Alzheimer's disease; BMD: bone mineral density; TB-BMD: total body bone mineral density; SNP, single nucleotide polymorphism; IVW, inverse variance weighted.

**Supplementary Figure 10:** (A-D) Funnel plot of the causal effect of BMD in different age groups on AD. (A) TB-BMD (age 0-15 years), (B) TB-BMD (age 30-45 years), (C) TB-BMD (age 45-60 years), and (D) TB-BMD (age over 60 years). (E-H) Leave-one-out analysis plots for BMD in different age groups on AD. (E) TB-BMD (age 0-15 years), (F) TB-BMD (age 30-45 years), (G) TB-BMD (age 45-60 years), and (H) TB-BMD (age over 60 years). AD: Alzheimer's disease; TB-BMD: total body bone mineral density.

**Supplementary Table 1.** Heterogeneity tests and directional horizontal pleiotropy test for the effect of AD on BMD at different sites.

**Supplementary Table 2.** Heterogeneity tests and directional horizontal pleiotropy test for the effect of AD on BMD in different age groups.

**Supplementary Table 3.** Heterogeneity tests and directional horizontal pleiotropy test for the effect of BMD at different sites on AD.

**Supplementary Table 4.** Heterogeneity tests and directional horizontal pleiotropy test for the effect of BMD in different age groups on AD.
